# Retraction Note: Intragastric exposure to titanium dioxide nanoparticles induced nephrotoxicity in mice, assessed by physiological and gene expression modifications

**DOI:** 10.1186/s12989-015-0097-1

**Published:** 2015-07-14

**Authors:** Suxin Gui, Xuezi Sang, Lei Zheng, Yuguan Ze, Xiaoyang Zhao, Lei Sheng, Qingqing Sun, Zhe Cheng, Jie Cheng, Renping Hu, Ling Wang, Fashui Hong, Meng Tang

**Affiliations:** Medical College of Soochow University, Suzhou, 215123 China; Key Laboratory of Environmental Medicine and Engineering, Ministry of Education, School of Public Health, Southeast University, Nanjing, 210009 China; Jiangsu key Laboratory for Biomaterials and Devices, Southeast University, Nanjing, 210009 China

## Retraction Note

This article [1] has been retracted by the Editor. A committee at Soochow University has investigated this case and supports the decision to retract the article. Incorrect statistical methods were used to calculate mean and S.D. values and additional errors were made in determining 8-OHdG concentrations. The committee also found that some of the original data were missing. We apologize to the readership of *Particle and Fibre Toxicology*.
